# Multimorbidity Analysis According to Sex and Age towards Cardiovascular Diseases of Adults in Northeast China

**DOI:** 10.1038/s41598-018-25561-y

**Published:** 2018-06-05

**Authors:** Lina Jin, Xin Guo, Jing Dou, Binghui Liu, Jiangzhou Wang, Jiagen Li, Mengzi Sun, Chong Sun, Yaqin Yu, Yan Yao

**Affiliations:** 1 School of Public Health, Jilin University, Changchun, Jilin 130021 China; 2 Key Laboratory for Applied Statistics of MOE and School of Mathematics and Statistics, Northeast Normal University, Changchun, Jilin 130024 China

## Abstract

Non-communicable diseases (NCDs) are great challenges in public health, where cardiovascular diseases (CVD) accounted for the large part of mortality that caused by NCDs. Multimorbidity is very common in NCDs especially in CVD, thus multimorbidity could make NCDs worse and bring heavy economic burden. This study aimed to explore the multimorbidity among adults, especially the important role of CVD that played in the entire multimorbidity networks. A total of 21435 participants aged 18–79 years old were recruited in Jilin province in 2012. Weighted networks were adopted to present the complex relationships of multimorbidity, and Charlson Comorbidity Index (CCI) was used to evaluate the burden of multimorbidity. The prevalence of CVD was 14.97%, where the prevalence in females was higher than that in males (*P* < 0.001), and the prevalences of CVD increased with age (from 2.22% to 38.38%). The prevalence of multimorbidity with CVD was 96.17%, and CVD could worsen the burden of multimorbidity. Multimorbidity and multimorbidity with CVD were more marked in females than those in males. And the prevalence of multimorbidity was the highest in the middle-age, while the prevalence of multimorbidity with CVD was the highest in the old population.

## Introduction

Non-communicable diseases (NCDs) are believed as leading causes of death in the world, and regarded as major threats to human health and sustainable development^1–3^. Of these, 17.60 million people died from cardiovascular diseases (CVD) in 2016^4^. In China, there were 230 million patients with CVD in 2010^5^, and the burden of CVD was also predicted to be a high level. Moreover, multimorbidity is very common in CVD, and it is reported that more than 50% CVD patients suffer from at least one additional disease^6,7^. Multimorbidity not only affects the quality of life among CVD patients, but also can bring heavy economic burden to individuals, families and the society^8^.

Many occurrences of multimorbidity with CVD had been recognized and investigated in previous studies, and hypertension was one of the most widely studied occurrences of multimorbidity with CVD^9–11^. World Health Organization (WHO) pointed out that approximately 13% of CVD deaths were accounted for hypertension^12^. Diabetes^13,14^, obesity^15^, and dyslipidemia^16,17^ were also recognized as the common occurrences of multimorbidity with CVD. Besides, some other diseases such as chronic respiratory disease^18^, liver disease^19^ and depression^20^ had been studied as potential occurrences of multimorbidity with CVD as well.

Moreover, some studies also showed that the pattern of multimorbidity of CVD was different among groups stratified by sex and age^6,21–23^. A study documented that the prevalence of anemia was the highest in female patients with heart failure, whereas the prevalence of dyslipidemia was the highest in male patients with heart failure^6^. Another study found that there were gender differences in the pattern of multimorbidity with CVD, and male patients were more likely to have multimorbidity^22^.

However, previous studies concerned only on one single occurrence of multimorbidity, rather than the overall occurrences of multimorbidity that covered information of multiple NCDs. In this study, we explored and presented the multimorbidity among adults in Jilin province, northeast China (Latitude 40°~46°, Longitude 121°~131°) in 2012, especially the important role of CVD that played in the entire multimorbidity networks. Weighted networks were adopted to demonstrate the complex relationships of co-occurrence of NCDs. The prevalences of multimorbidity and multimorbidity with CVD were more marked in females than those in males, and the prevalences were extremely high in the old population. We found that multimorbidity was extremely common among CVD patients, and CVD would also worsen the burden of multimorbidity.

## Results

### Sex-specific and age-specific distributions of top 20 NCDs of participants

Table 1 presents the top 20 NCDs with the highest prevalences, and the ranks of these diseases were a little different by sex and age. The prevalences of majority diseases were different by sex and age as well (*P* < 0.05). The prevalence of CVD was 14.49% (ranked the third), where in females it was higher than that in males, and the prevalences of CVD increased with age (from 2.22% to 38.38%).

**Table 1:** Sex-specific and age-specific distributions of top 20 NCDs of participants (n = 21435) in Northeast China [n (%)].

Rank	Disease	Total	Gender		*χ* ^2^	*P*-value	Age^a^			*χ* ^2^	*P*-value
			Male (n = 10337)	Female (n = 11098)			Young (n = 6657)	Middle-age (n = 12980)	Old (n = 1798)		
1	Hyperlipidemia	10951(51.09)	5379(52.04)	5572(50.21)	7.166	0.007	2288(34.37)	7504(57.81)	1159(64.46)	1108.124	<0.001
2	Hypertension	7511(35.04)	3893(37.66)	3618(32.60)	60.209	<0.001	892(13.40)	5470(42.14)	1149(63.90)	2315.335	<0.001
3	CVD	3209(14.97)	1213(11.73)	1996(17.99)	164.269	<0.001	148(2.22)	2371(18.27)	690(38.38)	1734.311	<0.001
4	Obesity	3105(14.49)	1529(14.79)	1576(14.20)	1.508	0.219	846(12.71)	2034(15.67)	225(12.51)	37.322	<0.001
5	Disc disease	2893(13.50)	1117(10.81)	1776(16.00)	123.814	<0.001	456(6.85)	2175(16.76)	262(14.57)	371.832	<0.001
6	Diabetes	1956(9.13)	972(9.40)	984(8.87)	1.859	0.173	143(2.15)	1488(11.46)	325(18.08)	650.085	<0.001
7	Rheumatoid arthritis	1942(9.06)	638(6.17)	1304(11.75)	202.102	<0.001	192(2.88)	1453(11.19)	297(16.52)	501.314	<0.001
8	Gastroenteritis	1923(8.97)	845(8.17)	1078(9.71)	15.521	<0.001	570(8.56)	1218(9.38)	135(7.51)	8.778	0.012
9	Cholecystitis	1585(7.39)	354(3.42)	1231(11.09)	459.494	<0.001	229(3.44)	1217(9.38)	139(7.73)	226.747	<0.001
10	Gynecological inflammation	1252(5.84)	0	1252(11.28)	—	—	460(14.92)	770(10.89)	22(2.38)	117.454	<0.001^b^
11	Gastric ulcer or duodenal ulcer	819(3.82)	427(4.13)	392(3.53)	5.219	0.022	164(2.46)	570(4.39)	85(4.73)	48.890	<0.001
12	Chronic bronchitis	767(3.58)	331(3.20)	436(3.93)	8.188	0.004	82(1.23)	544(4.19)	141(7.84)	215.102	<0.001
13	Prostate hyperplasia or inflammation	648(3.02)	648(6.27)	0	—	—	43(1.20)	464(7.85)	141(16.53)	334.018	<0.001^c^
14	Anemia	594(2.77)	72(0.70)	522(4.70)	318.934	<0.001	247(3.71)	309(2.38)	38(2.11)	32.030	<0.001
15	Fatty liver	510(2.38)	267(2.58)	243(2.19)	3.566	0.059	100(1.50)	377(2.90)	33(1.84)	39.753	<0.001
16	Calculus in urinary system	481(2.24)	271(2.62)	210(1.89)	12.981	<0.001	90(1.35)	348(2.68)	43(2.39)	35.629	<0.001
17	Rheumatic	442(2.06)	108(1.04)	334(3.01)	102.302	<0.001	63(0.95)	320(2.47)	59(3.28)	64.721	<0.001
18	Nasopharyngitis	439(2.05)	233(2.25)	206(1.86)	4.223	0.040	155(2.33)	257(1.98)	27(1.50)	5.583	0.061
19	Cataract	402(1.88)	113(1.09)	289(2.60)	66.392	<0.001	4(0.06)	219(1.69)	179(9.96)	759.591	<0.001
20	Gallstone	384(1.79)	125(1.21)	259(2.33)	38.466	<0.001	43(0.65)	284(2.19)	57(3.17)	80.677	<0.001

^a^The people with age less than 40 (age ≤ 40) were viewed as young, and people with 41 ≤ age ≤ 65 and age ≥ 66 were middle-age and old, respectively.

^b^The *χ*^2^ and the p-value were calculated among women only; ^c^the *χ*^2^ and the p value were calculated among men only.

### Sex-specific and age-specific top 5 patterns of multimorbidity

Table 2 shows the sex-specific and age-specific prevalences of top 5 patterns of multimorbidity, with the pair hyperlipidemia & hypertension as the highest one. Generally, the prevalences of CVD & hyperlipidemia and CVD & hypertension were also very high, especially in the old population.

**Table 2:** Sex-specific and age-specific top 5 patterns of multimorbidity [n (%)].

Group		Top 1	Top 2	Top 3	Top 4	Top 5
Total	—	Hyperlipidemia & Hypertension	Obesity & Hyperlipidemia	CVD & Hyperlipidemia	CVD & Hypertension	Obesity & Hypertension
		5015(23.40)	2183(10.18)	2065(9.63)	1913(8.92)	1759(8.21)
Sex	Male	Hyperlipidemia & Hypertension	Obesity & Hyperlipidemia	Obesity & Hypertension	CVD & Hypertension	CVD & Hyperlipidemia
		2564(24.80)	1101(10.65)	870(8.42)	782(7.57)	751(7.27)
	Female	Hyperlipidemia & Hypertension	CVD & Hyperlipidemia	CVD & Hypertension	Obesity & Hyperlipidemia	Hyperlipidemia & Disc disease
		2451(22.09)	1314(11.84)	1131(10.19)	1082(9.75)	1051(9.47)
Age^a^	Young	Hyperlipidemia & Hypertension	Obesity & Hyperlipidemia	Obesity & Hypertension	Hyperlipidemia & Gastroenteritis	Hyperlipidemia & Disc disease
		528(7.93)	519(7.80)	282(4.24)	211(3.17)	196(2.94)
	Middle-age	Hyperlipidemia & Hypertension	CVD & Hyperlipidemia	Obesity & Hyperlipidemia	CVD & Hypertension	Hyperlipidemia & Disc disease
		3708(28.57)	1543(11.89)	1488(11.46)	1380(10.63)	1321(10.18)
	Old	Hyperlipidemia & Hypertension	CVD & Hypertension	CVD & Hyperlipidemia	Diabetes & Hypertension	Diabetes & Hyperlipidemia
		779(45.58)	490(28.67)	472(27.62)	236(13.81)	232(13.58)

^a^The people with age less than 40 (age ≤ 40) were viewed as young, and people with 41 ≤ age ≤ 65 and age ≥ 66 were middle-age and old, respectively.

### Sex-specific and age-specific top 5 patterns of multimorbidity with CVD

Multimorbidity was extremely common among CVD patients, where there were 96.17% (3086/3209) CVD patients suffered from at least one other NCDs. Further, the prevalence of multimorbidity with CVD in females (97.29%) was more marked than that in males (94.31%), and it was the worst among old (97.54%) CVD patients (*P* < 0.001, 85.81% for the young and 96.42% for the middle-age). The top 5 patterns of sex-specific and age-specific multimorbidity with CVD were shown in Table 3. In general, the ranks for multimorbidity with CVD were similar, where hyperlipidemia and hypertension were the most frequent occurrences of multimorbidity among CVD patients, thus the “CVD-Hyperlipidemia-Hypertension” (CVD-H-H) triangle was inclined to play an important role in the multimorbidity networks.

**Table 3:** Sex-specific and age-specific top 5 patterns of multimorbidity among CVD patients [n (%)].

Group		Top 1	Top 2	Top 3	Top 4	Top 5
Total	—	Hyperlipidemia	Hypertension	Disc disease	Rheumatoid arthritis	Obesity
		2065(9.63)	1913(8.92)	806(3.76)	639(2.98)	611(2.85)
Sex	Male	Hypertension	Hyperlipidemia	Disc disease	Diabetes	Obesity
		782(7.57)	751(7.27)	235(2.27)	215(2.08)	214(2.07)
	Female	Hyperlipidemia	Hypertension	Disc disease	Rheumatoid arthritis	Obesity
		1314(11.84)	1131(10.19)	571(5.15)	479(4.32)	397(3.58)
Age^a^	Young	Hyperlipidemia	Hypertension	Disc disease	Gastroenteritis	Gynecological inflammation
		50(0.75)	43(0.62)	41(0.65)	29(0.44)	29(0.44)
	Middle-age	Hyperlipidemia	Hypertension	Disc disease	Obesity	Rheumatoid arthritis
		1543(11.89)	1380(10.63)	627(4.83)	479(3.69)	468(3.61)
	Old	Hypertension	Hyperlipidemia	Rheumatoid arthritis	Disc disease	Diabetes
		490(27.25)	472(26.25)	157(8.73)	138(7.68)	133(7.40)

^a^The people with age less than 40 (age ≤ 40) were viewed as young, and people with 41 ≤ age ≤ 65 and age ≥ 66 were middle-age and old, respectively.

### Evaluation of multimorbidity and multimorbidity networks

**Figure 1:**
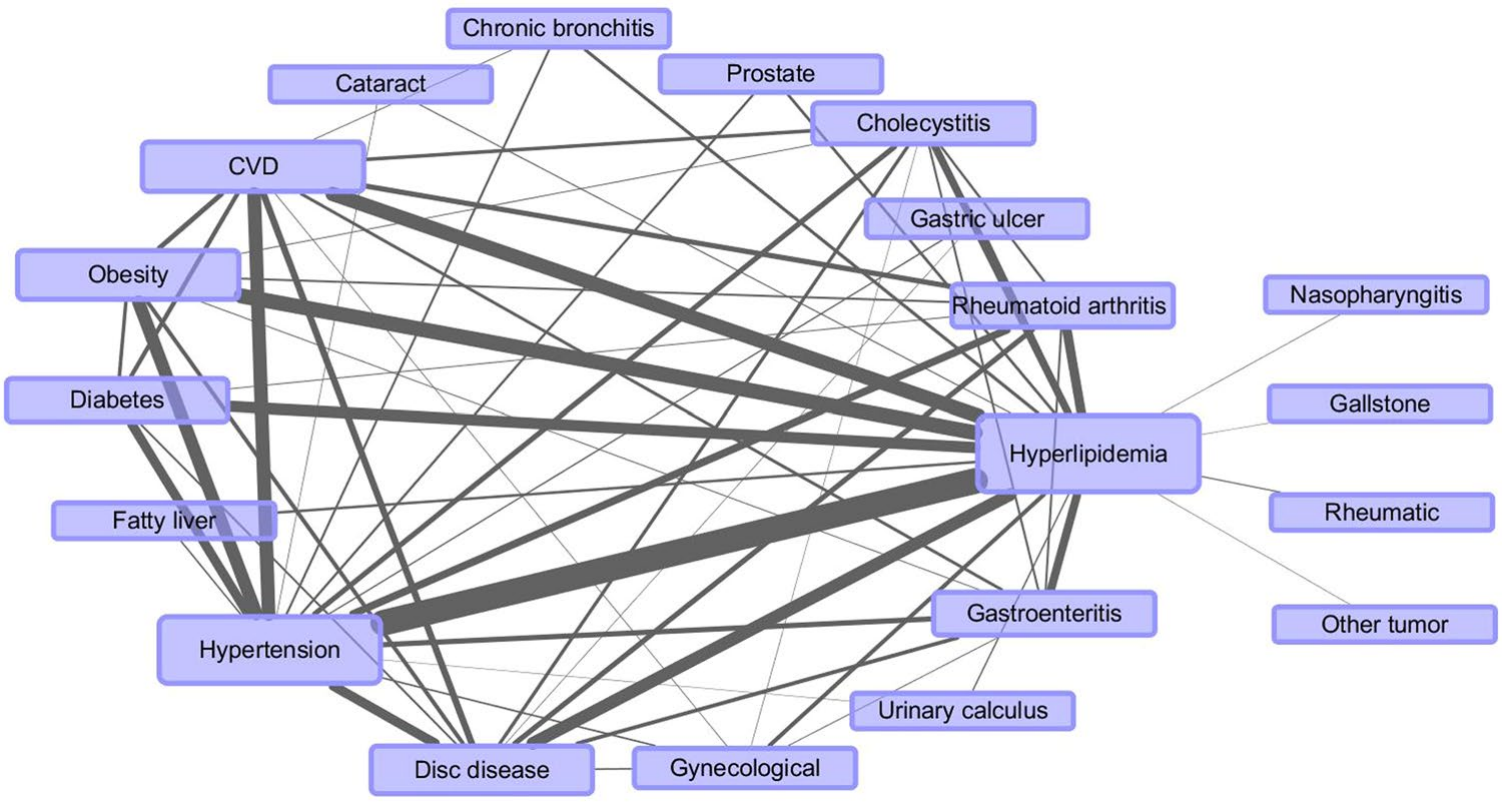
Multimorbidity network for the whole population, where “Prostate” represented “Prostate hyperplasia or inflammation”, “Gynecological” represented gynecological inflammation, “Gastric ulcer” represented gastric ulcer or duodenal ulcer; “other tumor” represented tumors except 9 cancer like liver cancer, lung cancer, etc.

**Figure 2:**
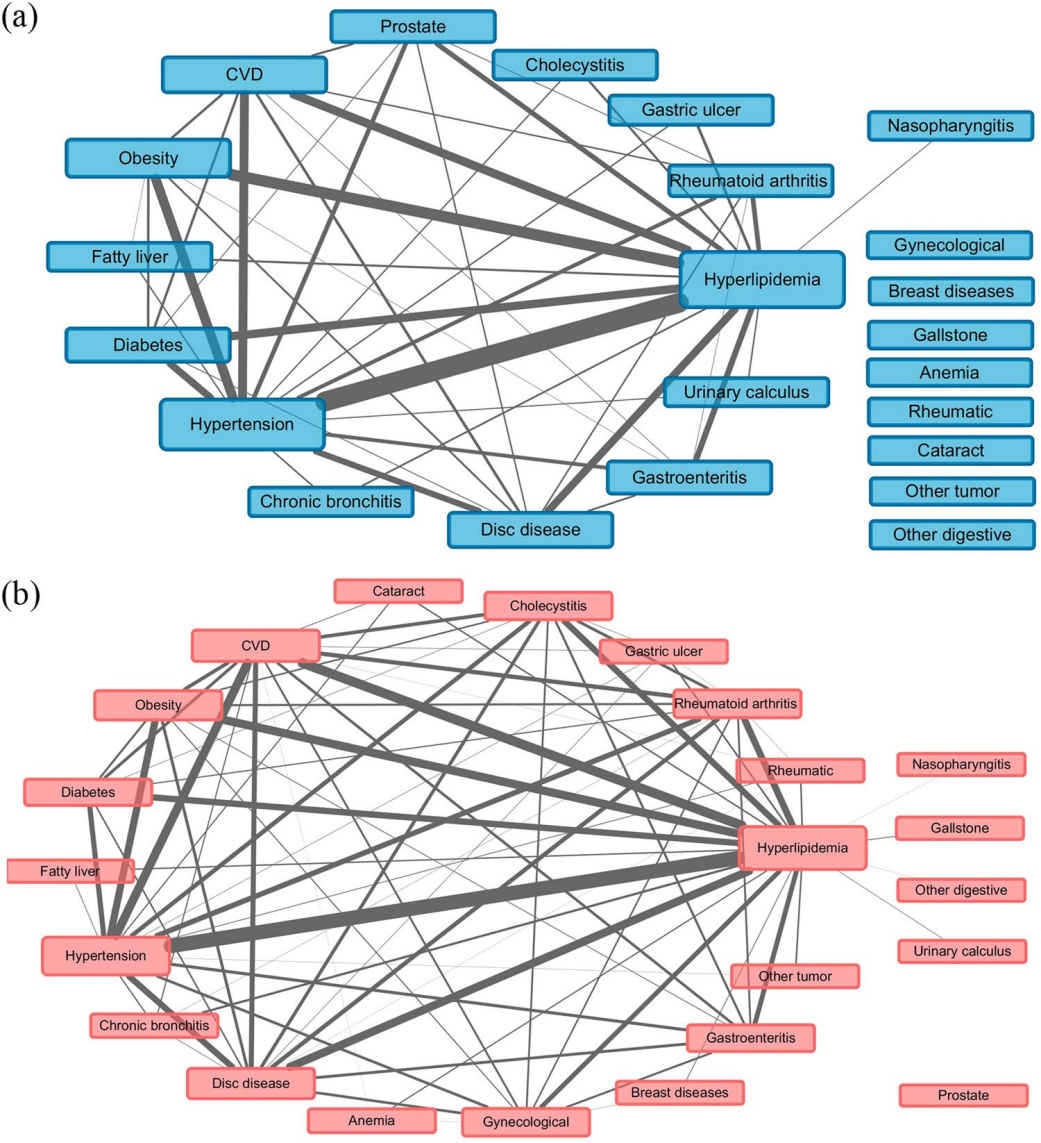
Multimorbidity networks by sex, where (**a**) for males and (**b**) for females, “Prostate” represented “Prostate hyperplasia or inflammation”, “Gynecological” represented gynecological inflammation, “Gastric ulcer” represented gastric ulcer or duodenal ulcer; “other tumor” represented tumors except 9 cancer like liver cancer, lung cancer, etc., and “other digestive” represented other diseases of digestive system except 7 ones like fatty liver, cirrhosis, etc.

**Figure 3:**
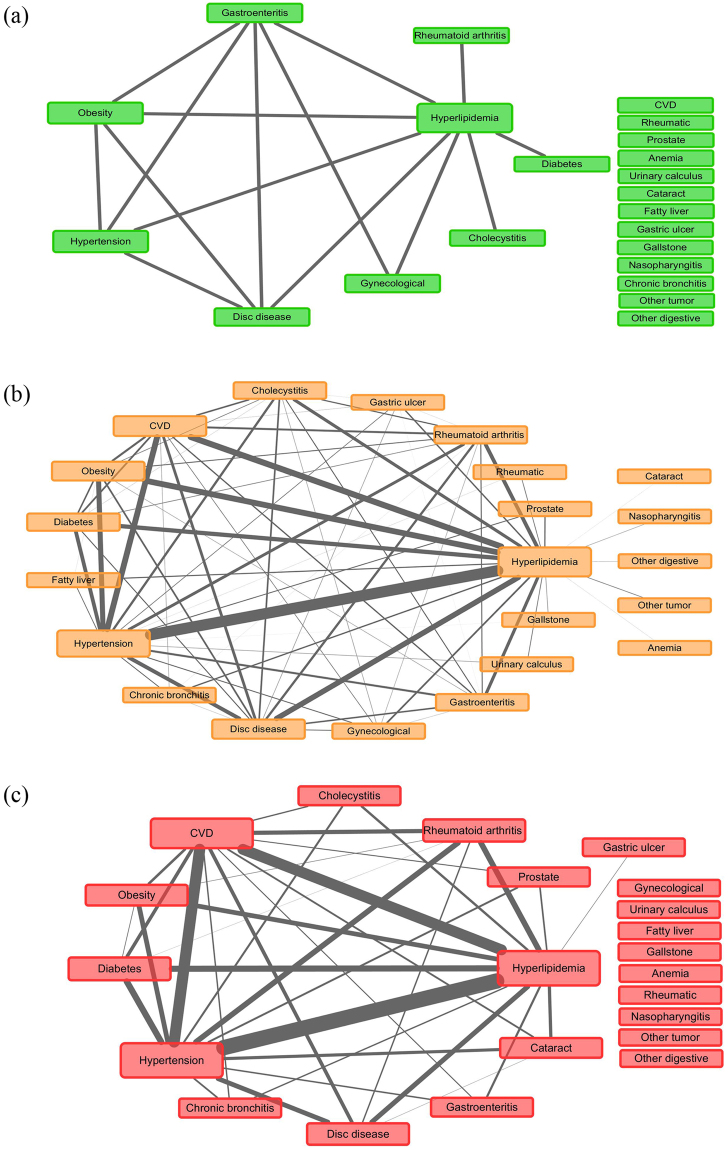
Multimorbidity networks by age group, where (**a**) for young (age ≤ 40), (**b**) for middle-age (41 ≤ age ≤ 65), and (**c**) for old (age ≥ 66), “Prostate” represented “Prostate hyperplasia or inflammation”, “Gynecological” represented gynecological inflammation, “Gastric ulcer” represented gastric ulcer or duodenal ulcer; “other tumor” represented tumors except 9 cancer like liver cancer, lung cancer, etc., and “other digestive” represented other diseases of digestive system except 7 ones like fatty liver, cirrhosis, etc.

Figures 1–3 showed the networks of the multimorbidity in the whole population, as well as the sex-specific and age-specific populations, and Table 4 list the indices which could measure the features of the networks. The network density and the average degree of females were larger than those of males, thus the network of females was much denser than that of males, i.e., the NCDs in females tended to co-occur more frequently than those in males. Meanwhile, the network density (as well as the average degree) reached the largest in the middle-age, and smallest in the young. In Table 4, each average degree of the CVD-H-H triangle was extremely higher than that of its network, where the average degree of the triangle in old population was 6.9 (22.67/3.27) times of its own network (Fig. 3(c)), which was the largest. Meanwhile, the proportion of CVD that contributed to the CVD-H-H triangle in old population was also the largest (11/(11 + 12 + 11) = 32.35%). The average degree of the CVD-H-H triangle in females was more marked than that in males, but compared with the corresponding female/male network, the CVD-H-H triangle in males was more important, which was 6.6 (24.67/3.74) times of its network (Fig. 2(b)). However, the proportion of CVD that contributed to the CVD-H-H triangle in males (8/(8 + 14 + 15) = 21.62%) was smaller than that in females (14/(14 + 21 + 13) = 29.17%).

**Table 4:** Evaluation of multimorbidity and multimorbidity networks.

Group		Network density	Average degree	Average degree of CVD-H-H^b^	CCI			
					CVD	non-CVD	*Z*	*P*-value
Total	—	0.305	5.80	29.33 (10, 19, 15)	3.80 ± 1.62	1.19 ± 1.34	72.26	<0.001
Sex	Male	0.170	3.74	24.67 (8, 14, 15)	3.71 ± 1.54	1.20 ± 1.31	45.41	<0.001
	Female	0.296	6.52	32.00 (14, 21, 13)	3.86 ± 1.66	1.18 ± 1.38	55.82	<0.001
Age^a^	Young	0.065	1.36	8.00 (0, 8, 4)	1.72 ± 1.03	0.29 ± 0.56	21.99	<0.001
	Middle-age	0.303	6.36	32.00 (11, 21, 16)	3.52 ± 1.44	1.48 ± 1.24	55.15	<0.001
	Old	0.156	3.27	22.67 (11, 12, 11)	5.22 ± 1.26	3.74 ± 1.08	23.09	<0.001

^a^The people with age less than 40 (age ≤ 40) were viewed as young, and people with 41 ≤ age ≤ 65 and age ≥ 66were middle-age and old, respectively.

^b^CVD-H-H refers to the “CVD-Hyperlipidemia-Hypertension” triangle in the network, and the values in the brackets are degrees of CVD, hyperlipidemia and hypertension.

Finally, the severity of the multimorbidity using Charlson Comorbidity Index (CCI) was also shown in Table 4. It was no surprising that the CCI in the population with CVD was extremely larger than that without CVD in all groups (P < 0.001), which indicated that CVD would bring extra burden to multimorbidity. Further, the CCIs in males were smaller than those in females (P = 0.013 for CVD and P = 0.002 for non-CVD), and CCIs were the highest among the elderly, and the lowest among the young (all *P* < 0.001).

## Discussion

NCDs are believed to bring great challenges to and have important impacts on public health nowadays, which accounted for 63% of deaths worldwide in 2008, and CVD is one of the most important main causes of deaths^24^. Meanwhile, multimorbidity was extremely common among the CVD patients^7,25^. In this study, we investigated the multimorbidity of 57 kinds of NCDs based on 21435 adults in Jilin province in 2012, especially the multimorbidity with CVD. Hyperlipidemia, hypertension and CVD were top 3 NCDs with the highest prevalences in Jilin province. Multimorbidity and the multimorbidity with CVD were more marked in females than those in males. The prevalence of multimorbidity was the highest in middle-age, whereas the prevalence of multimorbidity with CVD was the highest in the old population. 96.17% CVD patients suffered from multimorbidity, where the prevalence of multimorbidity increased with age, and CVD would worsen the burden of multimorbidity.

Hyperlipidemia, hypertension and CVD were top 3 NCDs with the highest prevalences in Jilin province, regardless of sex and age. The prevalence of CVD was 14.97%, which was lower than other studies in literature^5,26^, due to that hypertension was not included in CVD in this study. Although CVD ranked the third, the analysis of CCI suggested that CVD would bring extra burden to multimorbidity and increase the 10-year mortality^27^, thus the lethality and the burden of CVD with multimorbidity was much higher. Besides, among the top 5 patterns of multimorbidity there were 2 patterns of multimorbidity with CVD, which suggested that multimorbidity with CVD were very common. And 96.17% CVD patients suffered from multimorbidity, which was higher than other studies^22,23^, one possible reason might be that hyperlipidemia was investigated in our study.

Further, the CVD-H-H triangle in males was more marked than that in females, relative to their own network, but CVD in males contributed less proportions to the triangle than that in females. It was suggested that hyperlipidemia and hypertension in males played more important roles in multimorbidity, while CVD and its multimorbidity were more common in females, and would bring more risk to females^28,29^. Therefore, different strategies should be developed to prevent NCDs and their multimorbidity in males and females separately.

Finally, the prevalences of majority multimorbidity were the highest in the eldly, and the lowest in the young, which were consistent with other studies^30,31^. The possible reason might be that body immunity and function declined with age, so that the old people were more vulnerable to NCDs and their multimorbidity^32^. Although the middle-age had a denser multimorbidity network, the CVD-H-H triangle in the old population played a more important role, relative to their own network, where there CVD occupied large percentage compared with that of the young and middle-age. Thus it suggested different key prevention towards different age groups: multimorbidity with CVD were tended to cluster in the old population, while nutritional or metabolic diseases were common for young people^33^.

Some limitations should be noted here. Firstly, the participants in the study were selected in Jilin province, which could not represent the (CVD) multimorbidity in other places. Secondly, the disease situations were mainly based on self-report, which might cause bias. Thirdly, only cerebrovascular disorders, angina pectoris, coronary disease and myocardial infarction were involved in CVD, which might underestimate the prevalence of CVD and its multimorbidity. Finally, only sex and age were investigated in the study, but other factors that might have effects on the multimorbidity were worthy of further study.

## Methods

### Study population

Data were derived from a cross-sectional survey in Jilin Province of China in 2012, and the multistage stratified cluster sampling method was used to select the study samples. A total of 23050 participants who had lived in Jilin Province for more than 6 months and were 18–79 years old were selected (see more details in Part 1 of the Supplementary Material). For the purpose of the present analyses, some subjects were excluded due to missing values (1615 subjects). Finally, a total of 21435 subjects were included in the present analyses.

### Ethics Statement

The ethics committee of the School of Public Health, Jilin University (Reference Number: 2012-R-011) and the Bureau of Public Health of Jilin Province (Reference Number: 2012–10) approved the study. All research methods followed the guidelines of investigation and written informed consent was obtained from all of the participants before data collection.

### Data collection and measurement

The data of this study included demographics, anthropometric measurements (e.g., height, weight, blood pressure) and NCDs situations (57 NCDs, including liver cancer, lung cancer, gastric cancer, colorectal cancer, breast cancer, cervical cancer, prostate cancer, thyroid carcinoma, leukemia and other tumor (except the above 9 ones); anemia, rheumatic and other hematologic and immune related diseases (except the above 2); obesity, diabetes, hyperlipidemia, thyrotoxicosis, osteoporosis, gout and other endocrine, nutritional and metabolic diseases (except the above 6 ones); schizophrenia, depression and other mental & behavioral disorders (except the above 2); cognition disorders, epilepsy, Parkinson’s disease and other neurological diseases (except the above 3 ones); cataract, glaucoma and other eye diseases (except the above 2); hypertension, CVD (including cerebrovascular disorders, angina pectoris, coronary heart disease and myocardial infarction), corpulmonale, varicose veins of lower extremity and other diseases of circulatory system (except the above 4 ones); chronic obstructive pulmonary emphysema, asthma, nasopharyngitis, chronic bronchitis, and other respiratory diseases (except the above 4 ones); gastric ulcer or duodenal ulcer, fatty liver, cirrhosis, cholecystitis, gallstone, gastroenteritis, hernia of abdominal cavity, and other diseases of digestive system (except the above 7 ones); rheumatoid arthritis, disc disease, and other musculoskeletal and connective tissue diseases(except the above 2); nephritis, gynecological inflammation, breast diseases, urinary calculus, prostate hyperplasia or inflammation and other diseases of genitourinary system (except the above 5 ones)).

After 12 or more hours of overnight fasting, finger-tip blood samples were taken from the subjects, and the plasma glucose (FPG) level was analyzed; the 2-hour FPG level was also tested. These tests were conducted by a Bayer Bai Ankang fingertip blood glucose monitor machine. The serum lipids, including total cholesterol (TC), triglycerides (TG), high-density lipoprotein (HDL-C) and low-density lipoprotein (LDL-C), were measured before breakfast, using enzymatic methods in a central laboratory with standardized testing. Weight and height were performed after removing shoes and heavy outer clothing. Weights were measured to the nearest 0.1 kg using a calibrated scale with the subjects standing in an upright position, and heights were measured to the nearest 0.1 cm using a standard anthropometer. Body mass index (BMI) was calculated as weight/height^2^ (kg/m^2^). Blood pressure was measured using mercury sphygmomanometer in the sitting position after a 5-min rest period by trained professionals. Two readings each of systolic blood pressure (SBP) and diastolic blood pressure (DBP) were recorded, and the average of each measurement was used for data analysis. If the first two measurements differed by more than 5 mmHg, additional readings were taken^34–36^.

### Assessment criteria of disease

Hypertension was referred to those with SBP ≥ 140 mm Hg, and/or DBP ≥ 90 mm Hg, and/or normotensives treated with antihypertensive medications, and/or a self-reported history of hypertension^37^. Hyperlipidemia was defined as TC ≥ 5.18 mmol/L, and/or LDL-C ≥ 3.37 mmol/L, and/or HDL-C < 1.04 mmol/L, and/or TG ≥ 1.70 mmol/L, and/or normolipidemic subjects treated with antihyperlipidemia medications, and/or with history of hyperlipidemia diseases^38^. Obesity was defined as the BMI ≥ 28 kg/m^2 ^^39^. Diabetes was defined as FPG ≥ 7.0 mmol/l (126 mg/dl) and/or a self-reported history of diabetes. CVD was defined as a participant carried at least one of the following disease: cerebrovascular disorders, angina pectoris, coronary heart disease and myocardial infarction. Other NCDs were judged only by self-reported history of diseases which diagnosed in hospitals on county level and above.

### Statistical analysis

The continuous variables were expressed as means ± standard deviations (SD) and compared using the *t* test or Wilcoxon rank-sum test. The categorical variables were expressed as counts or percentages and compared using the Rao-Scott-*χ*^2^ test. All statistical analyses were performed with R version 3.4.1 (University of Auckland, Oakland, New Zealand). Statistical significance was set at a *P* value < 0.05.

In this study, weighted networks were applied to study the relationships among multimorbidity. The nodes of the network represented the diseases, and the height of each node was proportional to the prevalence of each disease. The edge in the network represented the co-occurrence of a multimorbidity pair, and the weight of the edge was proportional to the prevalence of each multimorbidity pair. When a participant carried more than 2 diseases, the count of every multimorbidity pair would have an increment of 1 (e.g., when a participant carried CVD, hypertension and hyperlipidemia, then the multimorbidity pair CVD & hypertension, hypertension & hyperlipidemia and hyperlipidemia & CVD would have an increment of 1). The prevalence of disease or multimorbidity pair were calculated as the total counts of participants which carried the disease or multimorbidity pair divided by the corresponding sample size. The relationships of the multimorbidity with prevalence higher than 1% were list in the networks in our study.

Degree was adopted to measure the centrality of a disease (e.g. CVD), where degree was the number of nodes that a focal node was connected to, which measured the involvement of the node in the network. Network density and average degree were used to evaluate the sparsity of a network. The network density of an undirected graph with *N* nodes and *M* edges was defined as 2 *M*/*N*(*N* − 1), which described the portion of the potential connections (*N*(*N* − 1)/2) in a network that were actual connections (*M*). The average degree was defined as the average of degrees of all nodes. The larger the network density (or average degree), the denser the network^40,41^. CCI was used to measure the burden of multimorbidity or comorbidity, which had been validated in many clinical settings to describe the condition of comorbidity and multimorbidity^42,43^. The larger the CCI, the worse the condition of multimorbidity (the larger 10-year mortality).

## Electronic supplementary material

Online Supplementary Material
