# Supplementary material for: Multimorbidity Analysis According to Sex and Age towards Cardiovascular Diseases of Adults in Northeast China
Source: Sci Rep. 2018 Jun 5;8:8607. doi: 10.1038/s41598-018-25561-y (PMC5988667; doi:10.1038/s41598-018-25561-y)
Supplement: Supplementary file 1 — Online Supplementary Material [file 41598_2018_25561_MOESM1_ESM.doc]

**Online Supplementary Material to**

**Multimorbidity Analysis According to Sex and Age towards Cardiovascular Diseases of Adults in Northeast China**

**Lina Jin^1^, Xin Guo^1^, Jing Dou^1^,** **Binghui Liu^2^, Jiangzhou Wang^2^, Jiagen Li^1^, Mengzi Sun^1^, Chong Sun^1^, Yaqin Yu^1^, Yan Yao ***

**1** School of Public Health, Jilin University, Changchun, Jilin, China, **2** Key Laboratory for Applied Statistics of MOE and School of Mathematics and Statistics, Northeast Normal University, Changchun, Jilin, China

* [yaoyan@jlu.edu.cn](mailto:yaoyan@jlu.edu.cn)

**1. Sampling Method**

Five-stage stratified random cluster sampling was used to select the samples under study. In the first stage, 32 districts/counties were identified in proportion to population, geographic location and ethnicity, from nine cities (Changchun, Jilin, Siping, Liaoyuan, Tonghua, Baishan, Songyuan, Baicheng and Yanbian). At the second stage, three or four towns (depending on the size of the district) were selected by stratified random sampling to guarantee the representativeness of each sample. In the third stage, three neighborhood committees were chosen by stratified random sampling from each of the towns previously selected. In the fourth stage, one village from each chosen neighborhood committee was selected by simple random sampling. In the final stage, cluster random sampling was used to identify individuals aged 18 to 79 years old from each of the villages selected for the study.
